# High Resolution Ion Mobility Enables the Separation of α-Galactose Containing Glycans from Their Non-α-Galactose Isomers

**DOI:** 10.7171/001c.165893

**Published:** 2026-09-01

**Authors:** Kimmy Park, Ron Orlando

**Affiliations:** 1 Complex Carbohydrate Research Center University of Georgia; 2 Department of Chemistry University of Georgia; 3 GlycoScientific, Athens, GA, 30602; 4 Department of Biochemistry and Molecular Biology University of Georgia

**Keywords:** Ion Mobility, HILIC, LC-MS, N-linked Glycans, α-Gal, immunogenic, alpha-galactose

## Abstract

Severe allergic reactions can occur when biotherapeutics containing the carbohydrate antigen galactose-α-1,3-galactose (α-Gal) are administered. The detection of α-Gal-containing N-glycans is a challenging task due to the presence of non-α-Gal-containing isomers. This study evaluates the ability of different analytical approaches to detect an α-Gal glycan in the presence of a non-α-Gal isomer. Cetuximab is known to contain N-glycans bearing the α-Gal epitope and was chosen for the study. N-glycans released from Cetuximab were analyzed using the HILIC-MS/MS system. HILIC-MS/MS successfully separated and identified α-Gal species when the Galactose number exceeded the antenna number. However, this system could not identify α-Gal species when the Galactose number equals the antenna number. A High-Resolution Ion Mobility Spectrometer (IMS) was also evaluated and found to resolve isomeric α-gal/non-α-gal isomeric glycans. The study demonstrates the utility of the IMS-MS system by detecting immunogenic glycans in the presence of their non-α-Gal isomers. To our knowledge, this is the first time this specific immunogenic pair of glycan isomers has been resolved in a biotherapeutic, which was enabled by a high-resolution IMS separation.

## Introduction

Adverse consequences are observed in patients receiving biotherapeutics, such as monoclonal antibodies (mAbs), that contain non-human glycans.^1–4^ An example is the administration of biotherapeutic products that carry Gal-α1-3Gal (α-Gal) glycans, which can lead to severe allergic reactions because humans lack an enzyme to synthesize α-Gal-containing glycans. The first report of α-Gal immunogenicity was a clinical study among patients who were administered Cetuximab for treatment of metastatic colorectal cancer or squamous-cell carcinoma of the head/neck.^5^ The study found that a novel Immunoglobulin E antibody (Ab) directed against α-Gal glycans in Cetuximab put patients at risk of hypersensitivity reactions.^5^

Detecting α-Gal glycans is a challenging task that requires high-resolution analytical methods. The structures of α-Gal and non-α-Gal N-glycans can be described using the following notation system: Ax represents the number of antennae, Gy is the number of Galactoses attached to antenna N-Acetylglucosamine (GlcNAc), Fz is the number of Fucoses linked to the core GlcNAc (Fz), and the number of existing α-Gal linkages is expressed as (α-Gal)w. An example of the N-glycan notation describing a species containing two antennae, one Galactose attached to antenna GlcNAc, one core Fucose, and one α-Gal linkage is A2G1(α-Gal)1F. Glycans with α-Gal linkage can often be found in mixtures with their non-α-Gal isomers, making the identification process difficult. For example, A2G2F is a common N-glycan found on biotherapeutic products and can co-exist with its α-Gal isomer called A2G1(α-Gal)1F. Mass spectrometry (MS) is a highly sensitive tool that has been coupled with separational techniques to enhance the detection of α-Gal glycans in biotherapeutics.^6–9^ Capillary electrophoresis (CE) is a common tool employed in glycan separation^10^ and was paired with electrospray ionization (ESI) MS in a characterization study of α-Gal-containing glycans in beef, mutton, and pork tenderloin.^11^ Although CE-MS successfully provided a profile of α-Gal species found in the tested animal samples, the separation of isomers such as A2G1(α-Gal)1F and A2G2F was not achieved.^11^ Hydrophilic Interaction Liquid Chromatography (HILIC) is another popular separation technique in glycomics^12^ that was combined with a Fluorescence Detector (FLD) and MS for glycoprofilling of Cetuximab expressed from different sources.^13^ HILIC separation provided a detailed characterization of N-glycan species in Cetuximab. Still, this method fails to separate α-Gal-containing glycans from their non-α-Gal isomers due to the reported coelution of A2G1(α-Gal)1F with A2G2F, A3G1(α-Gal)1F with A3G2F, and A2G1(α-Gal)1F2 with A2G2F2.^13^ Developing an analytical method with high resolving power remains a crucial need in distinguishing α-Gal glycans from their non-α-Gal isomers.

Ion mobility spectrometry (IMS) is gaining attention as a powerful tool in glycomics studies.^14–17^ One of the first applications of ion mobility in carbohydrate characterization demonstrated different drift times for isomers present in a series of oligosaccharides.^14^ An ion mobility approach called Structure for Lossless Ion Manipulation (SLIM) provides higher resolving power because it uses a 13 m separation path, and thus can resolve components of complex isomeric mixtures that cannot be performed on ion mobility spectrometers with shorter paths.^18,19^ A separation of positional isomers (α1-3/α1-6) from A2G1 and A2G1F was achieved with G1(6) arriving earlier than G1(3) via the SLIM device,^20^ which suggests that SLIM may be a potential tool for separating α-Gal glycans from their non-α-Gal positional isomers.

This study evaluates the ability of different analytical methods to detect α-Gal N-glycans in biotherapeutic products. Cetuximab was chosen due to the high abundance of α-Gal N-glycans. HILIC combined with tandem mass spectrometry (MS/MS) was initially used for the analysis of N-glycans released from Cetuximab, but HILIC-MS/MS struggled to identify A2G1(α-Gal)1F from A2G2F. The sample was then run by HILIC-IMS-MS. IMS successfully separated A2G1(α-Gal)1F from A2G2F. Exoglycosidase digestions were performed to confirm the identity of N-glycan isomers following the observed separation. To our knowledge, this is the first time this specific immunogenic pair of glycan isomers has been resolved in a biotherapeutic, and we believe this separation was enabled by the high-resolution IMS separation provided by the SLIM device. These results demonstrate the power of HILIC-IMS-MS for the analysis of complex isomeric glycan mixtures.

## Materials and Methods

### Materials

Cetuximab was purchased from Eli Lilly (Indianapolis, IN, USA). Human Serum, Dextran from Leuconostoc spp (Mr 6000), procainamide hydrochloride, acetic acid, and dimethyl sulfoxide (DMSO) were purchased from Sigma-Aldrich (St. Louis, MO, USA). Sodium cyanoborohydride was purchased from Acros Organics (Branchburg, NJ, USA). PNGaseF was purchased from Lectenz Bio (Athens, GA, USA). Denaturing buffer and NP40 were purchased from New England Biolabs (Ipswich, MA, USA). PD MiniTrap G10 and Hi-Trap™ protein G HP column were purchased from Cytiva (Marlborough, MA, USA). HyperSep^TM^ C18 Cartridge was purchased from ThermoFisher (Waltham, MA, USA). β1-3,4 Galactosidase (cloned from bovine testis) and α1-3,4,6 Galactosidase (cloned from green coffee bean and expressed in *E. coli*) were purchased from New England Biolabs (Ipswich, MA, USA). Ammonium bicarbonate, ammonium formate, and formic acid were purchased from Fluka (Morris Plains, NJ, USA). Acetonitrile (LC-MS graded) was purchased from Honeywell Burdick and Jackson (Muskegon, MI, USA). Adalimumab was purchased from GlycoScientific (Athens, GA, USA).

### Release of N-glycans from Protein/Antibody

Enzymatic deglycosylation was performed according to a previously described protocol.^21^ Briefly, human Serum Immunoglobulin Gs (IgGs) were purified from human serum using a Hi-Trap™ protein G HP column before analysis. 1 mg of Cetuximab, 1 mg of Adalimumab (human IgG1), and 1 mg of Human Serum IgG were denatured at 100°C for 12 minutes using 4 µL of denaturing buffer (40 mM DTT, 0.5% sodium dodecyl sulfate [SDS]). The denatured proteins were placed in the freezer for 5 minutes, and 4 µL of 1% NP-40 was added to prevent PNGaseF deactivation from residual heat and the inhibitory effect of SDS. Then, 4 µL of PNGaseF was added to the sample, and the mixture was incubated at 37°C. After 16–20 hours, the samples were collected and lyophilized to dryness.

### N-glycan Purification and Procainamide (ProA) Labeling

Released N-glycans were labeled with procainamide according to a previously described protocol.^22^ Briefly, the released N-glycans were resuspended in 200 µL of 5% acetic acid and run through a C18 SPE column (conditioned with 6 mL of 100% methanol and calibrated with 6 mL of 5% acetic acid) to remove residual protein(s). The purified N-glycans were collected and lyophilized to dryness. The labeling solution was prepared with 216 mg/mL procainamide hydrochloride and 126 mg/mL sodium cyanoborohydride in a 7:3 (DMSO/acetic acid) solution. Approximately 200 µL of the labeling solution was added to the purified N-glycans. The mixtures were incubated at 37°C overnight. After incubation, 100 µL of acetone was added to quench the reaction at 65°C for 30–60 minutes. The samples were dried down and resuspended in 140–200 µL of 5% acetic acid. Then, excess labeling reagents were cleaned up using a size-exclusion PD MiniTrap G10 column. The collected fractions were lyophilized and stored in 100–150 µL of 80% ACN/H_2_O at -20°C until analysis.

### Exoglycosidase Digestions

To confirm the existence of α-Gal and β-Gal linkages, 30 µL of N-glycans solution released from Cetuximab was divided into two vials (15 µL each) and dried down. The first vial was digested with 5 µL of β1-3,4 Galactosidase (8000 U/mL) and 5 µL of GlycoBuffer (as received) in 45 µL of nanopure water. The second vial was digested with 5 µL of α1-3,4,6 Galactosidase (8000 U/mL), 5 µL purified BSA (as received), and 5 µL GlycoBuffer 1 (as received) in 40 µL nanopure water. The digestions were incubated at 37°C overnight. After digestion, each reaction solution was lyophilized to dryness and then resuspended in 80% ACN/H_2_O for analysis.

### HILIC-MS/MS Settings and Instrumentations

The samples were run on an LC-MS system consisting of an Agilent 1100 (Santa Clara, CA) interfaced to a Waters Synapt G2 (Milford, MA). A Halo Penta-HILIC column (Advanced Material Technology, 1.5 mm x 15 cm, 2.7 µm particle size, Wilmington, DE) was used for the separation, which was performed at 0.1 mL/min flow rate at 60°C with mobile phase A and B consisting of 100% ACN with 0.1% formic acid and 50 mM ammonium formate in water with 0.1% formic acid, respectively. The gradient started at 80% ACN and ramped down 40% ACN over 40 minutes. The mass spectrometer operated in data-dependent mode, in which the five most intense ions from each full mass spectrum were selected for fragmentation via collision-induced dissociation. Data was analyzed via Waters MassLynx and Skyline. N-glycan annotations were built by GlycoWorkbench version 1.1 build 3480.

### HILIC-IMS-MS Settings and Instrumentations

The samples were run on an LC-MS system consisting of an Agilent 1290 Infinity II LC–MOBILion SLIM–Agilent 6546 Q-TOF (MOBILion Systems Inc., Chadds Ford, PA & Agilent, Santa Clara, CA). The HILIC separation was achieved using a Halo Penta-HILIC column (Advanced Material Technology, 1.5 x 150 mm, 2.7 µm particle size, Wilmington, DE). The separation was performed at a flow rate of 0.1 mL/min at 60°C using mobile phases A and B: 100% ACN with 0.1% formic acid and 50 mM ammonium formate in water with 0.1% formic acid, respectively. The gradient started at 80% ACN and ramped down to 40% ACN over 40 minutes.

The analytes were introduced into the IMS-MS system after HILIC separation. Ions were generated using the Dual AJS ESI from Agilent (Santa Clara, CA) with the recommended settings for N-glycan ionization.^23^ Upon entering the SLIM module, the ions were filled in the storage section and then released into a single-pass path length of 13 m.^24–26^ The Traveling Wave (TW) potentials were generated between the electrodes on two parallel printed circuit boards to propel ions through N_2_ drift gas.^24–26^ The settings for the TW potentials were based on the provider’s recommended parameters (MOBILion Systems Inc., Chadds Ford, PA), with minimal optimization. The mass spectra were obtained using MS (seq) acquisition settings in the Agilent 6546 Q-TOF (Agilent, Santa Clara, CA).

The collected data were processed via the HRIM Data Processor prior to analysis using Agilent MassHunter IM-MS Browser and Skyline. Procainamide-labeled dextran was used as the external calibrant for collision cross section (CCS) calculations. CCS values of procainamide-labeled dextran were extracted from the reference data of Manz et al.^27^ The CCS calculation was based on the method described by Li et al.^28^

## Results

### Challenges in Detecting α-Gal-Containing N-Glycans

**Figure 1.**
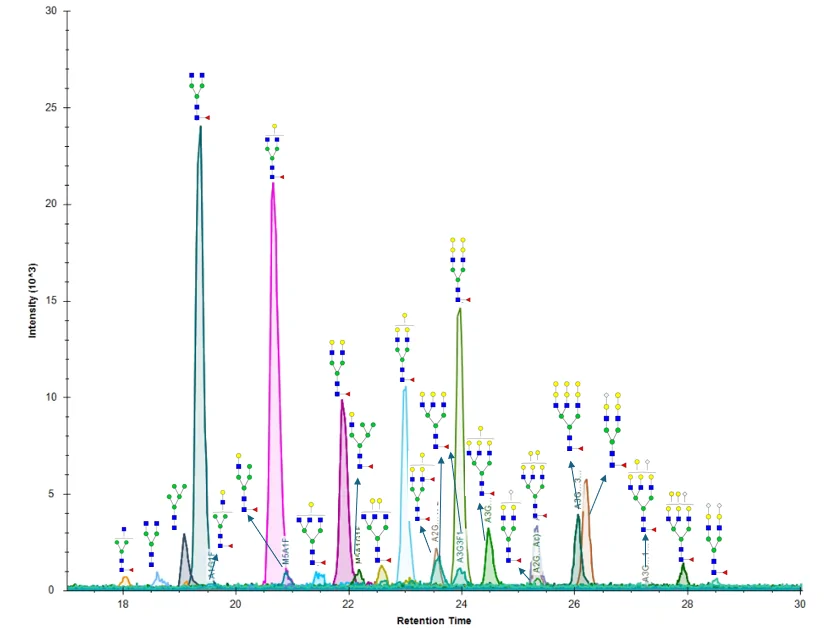
**HILIC analysis of major N-glycans released from Cetuximab.** HILIC can easily resolve α-Gal glycans that have a greater Galactose number than the antenna number, such as A2G2(α-Gal)1F, A2G2(α-Gal)2F, A3G3(α-Gal)1F, A3G3(α-Gal)2F, and A3G3(α-Gal)3F.

**Figure 2.**
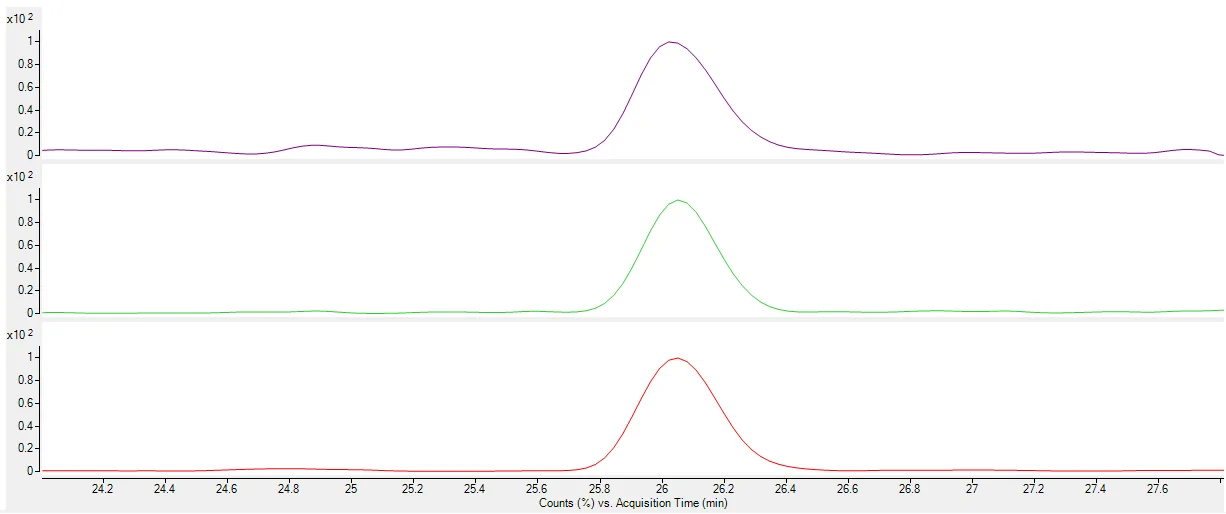
**Extracted ion chromatograms (EICs) of m/z = 1003.9 from the β1-3,4 Galactosidase digested Cetuximab (A), the α1-3,4,6 Galactosidase digested Cetuximab (B), and standard Cetuximab (C).** The m/z = 1003.9 is the doubly protonated mass to charge of procainamide-labeled A2G2F2 or A2G2(α-Gal)1F. The similarity in retention times of these EICs indicates that HILIC-MS is unable to resolve A2G2(α-Gal)1F and A2G2F.

The identification of α-Gal-containing glycans is crucial for ensuring safety in drug development. This analysis is challenging in the presence of non-α-Gal isomers. Cetuximab is known to contain a variety of α-Gal glycans, such as A2G2(α-Gal)1F or A2G1(α-Gal)1F, and was used as the positive control. Human Serum IgG and Adalimumab (IgG1) do not contain α-Gal glycans and were chosen as negative controls. The detection of α-Gal glycans can be easily achieved using HILIC-MS when the Galactose number is greater than the antenna number, i.e., A2G2(α-Gal)1F, A2G2(α-Gal)2F, A3G3(α-Gal)1F, A3G3(α-Gal)2F, A3G3(α-Gal)3F (Figure 1). However, detection becomes significantly more challenging when the number of Galactose is equal to or less than the antenna number, i.e., A2G1(α-Gal)1F vs A2G2F. The HILIC-MS analysis shows that HILIC is unable to resolve A2G1(α-Gal)1F and A2G2F (Figure 2).

**Figure 3.**
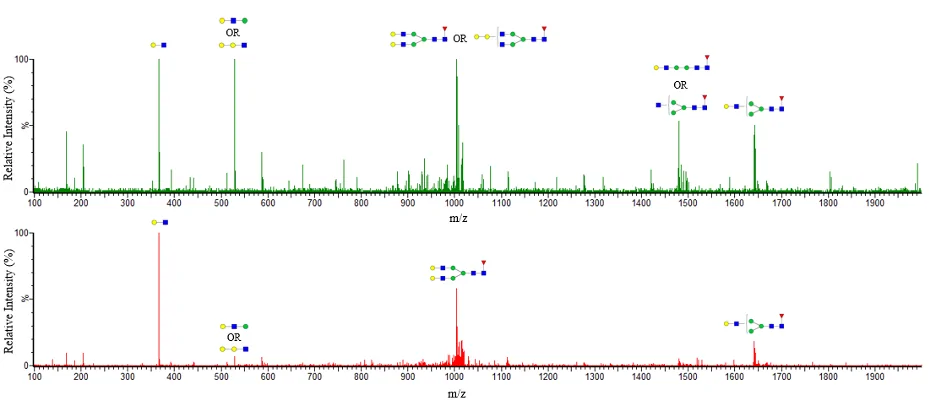
**The tandem mass spectrum of the precursor ion at m/z = 1003.9 from Cetuximab (A) and Human Serum IgG (B).** It is worth noting that the abundance of the fragment ion at 528 m/z is significantly higher in (A).

The behavior of α-Gal glycans was studied using MS/MS. Human serum IgG is a non-α-Gal source and was chosen as one of the negative controls for the study. The abundance of (HexNAc)_1_(Hexose)_2_ (m/z 528) fragment ion was noticeably high in the MS/MS spectrum of 1003.9 m/z (A2G2F/A2G2(α-Gal)1F) Cetuximab (Figure 3A). The m/z 528 fragment ion could be indicated as either GalGalGlcNAc or GalGlcNAcMan; hence, the non-α-Gal species can produce a fragment ion at this m/z value (Figure 3B). However, the abundance of the 528 m/z fragment ion is expected to vary with the ratio of α-Gal to non-α-Gal species, thus limiting the dynamic range for detection of α-Gal in the presence of its non-α-Gal isomer.

### Analysis via HILIC- IMS-MS

HILIC-MS/MS failed to detect A2G1(α-Gal)1F in the presence of A2G2F, which is one of the most common non-α-Gal N-glycans found in antibodies. A secondary separation dimension (High-Resolution Ion Mobility - HRIM) was added to increase the system’s resolving power, enabling the identification of α-Gal N-glycans in the presence of their non-α-Gal isomers.

**Figure 4.**
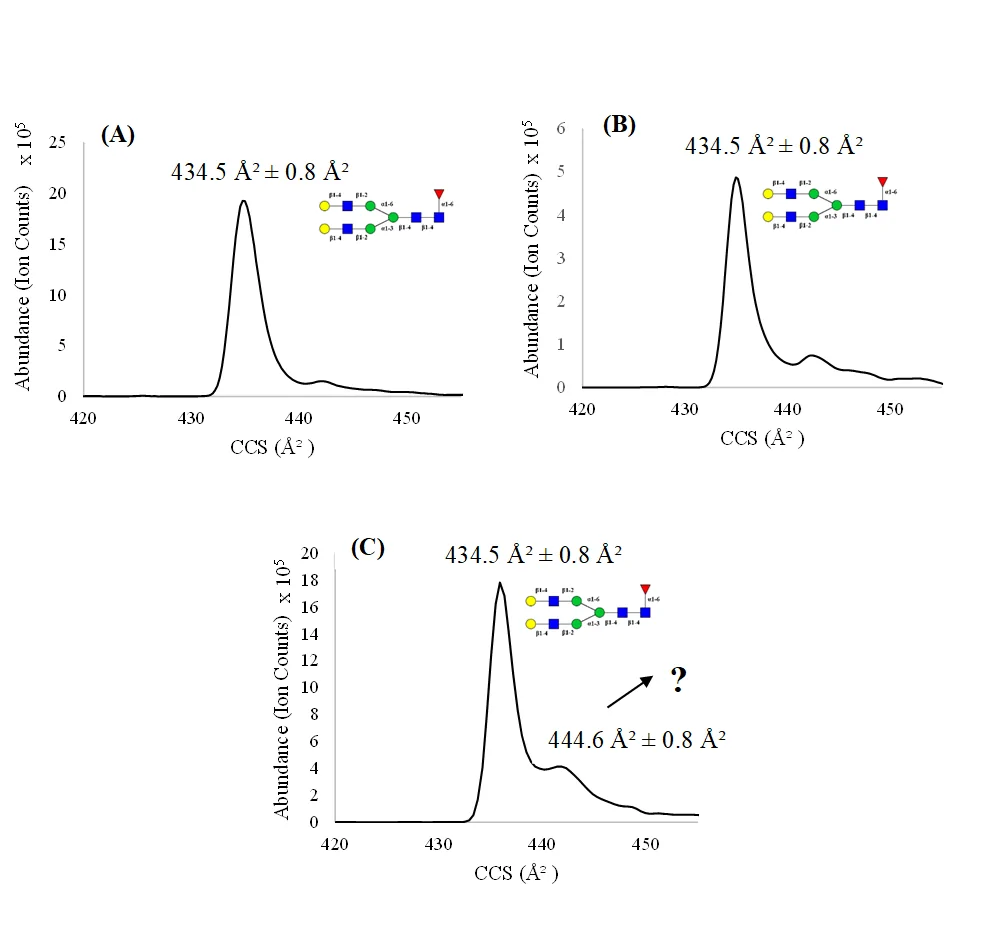
**The mobiligrams of m/z = 1003.9 from (A) Adalimumab (IgG1), (B) Human Serum IgG, and (C) Cetuximab.** The m/z = 1003.9 (M+2H) is the mass to charge of procainamide-labeled A2G2F2 or A2G2(α-Gal)1F. Both mobiligrams collected from negative controls (IgG1 and Human Serum IgG) show a single peak at 434.5 Å², confirming that A2G2F has a mobiligram trace with a single dominant peak with a CCS value of 434.5 Å². An additional peak at 444.6 Å² was detected in the mobiligram of Cetuximab (C), suggesting the presence of the α-Gal isomer.

Ion mobility traces of 1003.9 m/z (A2G2F) from Human Serum IgG and Adalimumab (IgG1) samples have a single peak with the CCS value of 434.5 Å² ± 0.8 Å² (Figure 4A and 4B). The mobiligram at 1003.9 m/z from the Cetuximab sample (Figure 4C) was collected, and two distinct peaks were observed with CCS values of 434.5 ± 0.8 Å² and 444.6 ± 0.8 Å², respectively. The peak at 434.5 Å² from Cetuximab mobiligram (Figure 4C) has a similar shape and identical CCS value as the peak found in both Human Serum IgG and IgG1 mobiligrams, suggesting that the 434.5 Å² peak results from the A2G2F species. The presence of the second peak at 444.6 Å² suggests that an isomer coelutes with the A2G2F species in HILIC separation. The peak at 444.6 Å² is hypothesized to be A2G1(α-Gal)1F since this was observed in the positive control (Cetuximab) but not in the sources that lack α-gal glycans.

### Exoglycosidase Digestions

**Figure 5.**
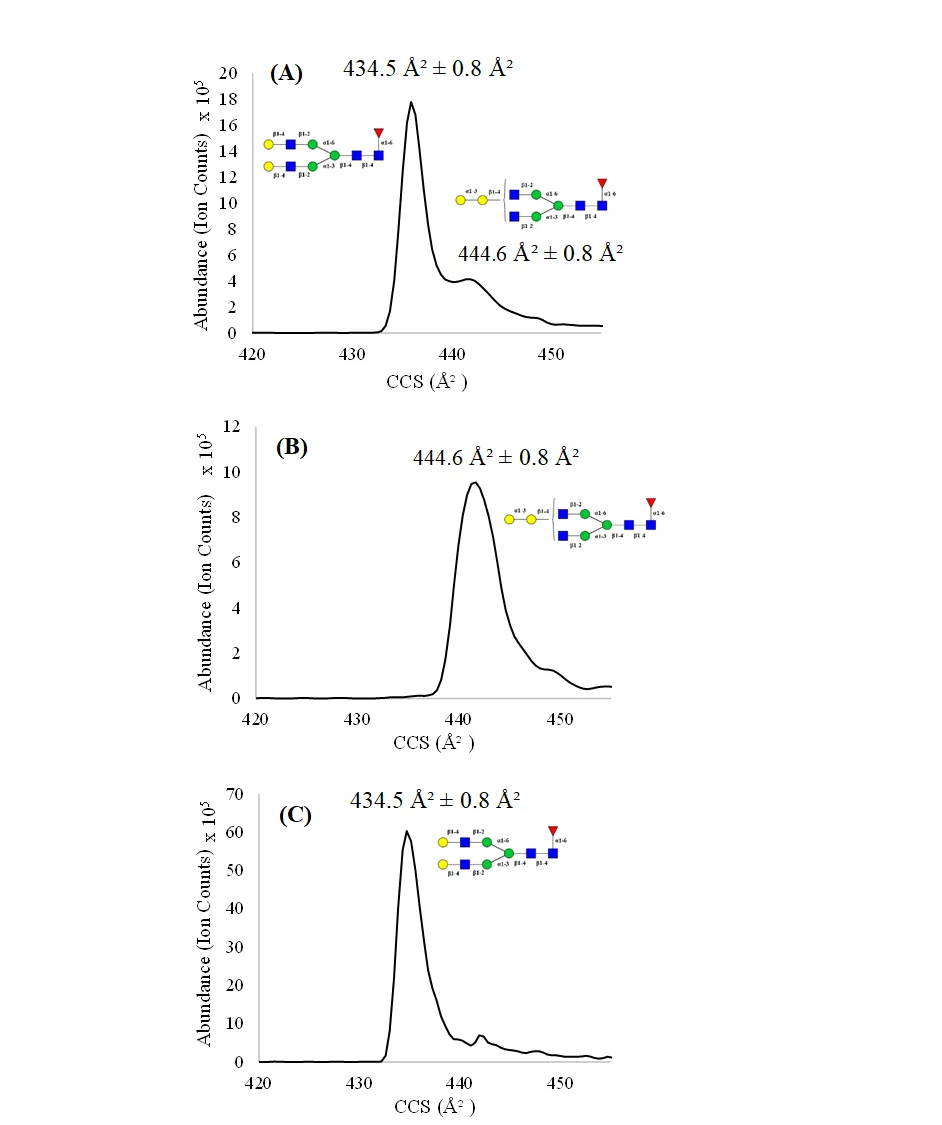
**HILIC-IMS-MS results showing the mobiligram traces of at the ion at 1003.9 m/z units from Cetuximab, obtained before (A) digestion, (B) after β1-3,4 Galactosidase digestion, and (C) after α1-3,4,6 Galactosidase digestion.** The disappearance of the peak at 434.5 Å² after β1-3,4 Galactosidase digestion demonstrates that both of the Gal residues are attached via a B-linkage. In comparison, the disappearance of the peak at 444.6 Å² after α1-3,4,6 Galactosidase digestion demonstrates that this species has a single Gal terminus, which is attached via an alpha linkage.

A series of exoglycosidase digestions was conducted to confirm the identity of the species with CCS values of 434.5 Å² and 444.6 Å in 1003.9 m/z mobiligram of Cetuximab (Figure 5A). N-glycans released from Cetuximab were treated with β1-3,4 Galactosidase. The disappearance of the peak at 434.5 Å² (Figure 5B) after β1-3,4 Galactosidase digestion indicates that β-linkages attach the Gal residues at the termini of this glycan. Hence, this species corresponds to the expected A2G2F structure. The peak at 444.6 Å² was not affected by the β1-3,4 Galactosidase digestion, suggesting the existence of an α-Gal residue, which blocks cleavage of β-linkage. A second experiment was conducted with α1-3,4,6 Galactosidase to confirm the presence of α-Gal species with a CCS value of 444.6 Å². The disappearance of this large peak after α-galactosidase digestion (Figure 5C) confirms the presence of an α-Gal linkage and identifies this as the A2G1(α-Gal)1F species. The exoglycosidase digestions revealed the ability of IMS to separate α-Gal glycans from their non-α-Gal isomers.

## Discussion

This study reveals that IMS can separate α-Gal glycans from their non-α-Gal isomers, a task that remains challenging in the biopharmaceutical industry. CE and high-performance liquid chromatography (HPLC) are popular separation methods that have been applied to analyze glycans,^10,12^ but these traditional systems are incapable of resolving some challenging isomers.^11,13^ The ion mobility traces from non-α-Gal controls were compared with the positive α-Gal control and confirmed the presence of an unknown co-eluting glycan species that HILIC could not separate. Exoglycosidase digestions were performed and demonstrated the presence of an α-Gal-containing glycan at the second peak at 444.6 Å², which was identified as the A2G1(α-Gal)1F. While the results focus on a single particularly important isomeric pair [A2G1(α-Gal)1F vs A2G2F], similar CCS separations are expected for other α-Gal /non-α-Gal pairs (e.g., higher antenna structures); however, these larger glycans may require higher resolution IMS separations.

The HILIC-IMS-MS method used in this paper not only enables the separation of α-Gal isomers but also provides three-dimensional data for detailed analysis of complex N-glycans from an antibody. Future studies utilizing HILIC-IMS-MS can significantly benefit from both HILIC retention-time data and HRIM data, thereby enabling high efficiency and accuracy in data analysis.

### Conflict of Interest

Ron Orlando is a founder/owner of GlycoScientific, Athens, Georgia, USA. Adalimumab used in this study was obtained from GlycoScientific. The authors declare no other competing financial interests.
